# Targeting Myeloid-Derived Suppressor Cells to Enhance a Trans-Sialidase-Based Vaccine Against *Trypanosoma cruzi*

**DOI:** 10.3389/fcimb.2021.671104

**Published:** 2021-07-06

**Authors:** Juan Cruz Gamba, Carolina Roldán, Estefanía Prochetto, Giuliana Lupi, Iván Bontempi, Carolina Verónica Poncini, Mónica Vermeulen, Ana Rosa Pérez, Iván Marcipar, Gabriel Cabrera

**Affiliations:** ^1^ Facultad de Bioquímica y Ciencias Biológicas, Universidad Nacional del Litoral, Santa Fe, Argentina; ^2^ Facultad de Ciencias Médicas, Universidad Nacional del Litoral, Santa Fe, Argentina; ^3^ Departamento de Microbiología, Parasitología e Inmunología, Facultad de Medicina, Universidad de Buenos Aires, Buenos Aires, Argentina; ^4^ Laboratorio de Inmunología Oncológica, Instituto de Medicina Experimental (IMEX-CONICET), Academia Nacional de Medicina, Buenos Aires, Argentina; ^5^ IDICER-CONICET and Instituto de Inmunología, Facultad de Ciencias Médicas, Universidad Nacional de Rosario, Santa Fe, Argentina

**Keywords:** *Trypanosoma cruzi*, myeloid derived suppressor cells, vaccine, trans-sialidase, adjuvant

## Abstract

*Trypanosoma cruzi* (*T. cruzi*) is a hemoflagellate protozoan parasite that causes Chagas disease, a neglected tropical disease that affects more than 6 million people around the world, mostly in Latin America. Despite intensive research, there is no vaccine available; therefore, new approaches are needed to further improve vaccine efficacy. It is well established that experimental *T. cruzi* infection induces a marked immunosuppressed state, which includes notably increases of CD11b+ GR-1+ myeloid-derived suppressor cells (MDSCs) in the spleen, liver and heart of infected mice. We previously showed that a trans-sialidase based vaccine (TSf-ISPA) is able to confer protection against a virulent *T. cruzi* strain, stimulating the effector immune response and decreasing CD11b+ GR-1+ splenocytes significantly. Here, we show that even in the immunological context elicited by the TSf-ISPA vaccine, the remaining MDSCs are still able to influence several immune populations. Depletion of MDSCs with 5 fluorouracil (5FU) at day 15 post-infection notably reshaped the immune response, as evidenced by flow cytometry of spleen cells obtained from mice after 21 days post-infection. After infection, TSf-ISPA-vaccinated and 5FU-treated mice showed a marked increase of the CD8 response, which included an increased expression of CD107a and CD44 markers in CD8+ cultured splenocytes. In addition, vaccinated and MDSC depleted mice showed an increase in the percentage and number of CD4+ Foxp3+ regulatory T cells (Tregs) as well as in the expression of Foxp3+ in CD4+ splenocytes. Furthermore, depletion of MDSCs also caused changes in the percentage and number of CD11c^high^ CD8α+ dendritic cells as well as in activation/maturation markers such as CD80, CD40 and MHCII. Thus, the obtained results suggest that MDSCs not only play a role suppressing the effector response during *T. cruzi* infection, but also strongly modulate the immune response in vaccinated mice, even when the vaccine formulation has a significant protective capacity. Although MDSC depletion at day 15 post-infection did not ameliorated survival or parasitemia levels, depletion of MDSCs during the first week of infection caused a beneficial trend in parasitemia and mice survival of vaccinated mice, supporting the possibility to target MDSCs from different approaches to enhance vaccine efficacy. Finally, since we previously showed that TSf-ISPA immunization causes a slight but significant increase of CD11b+ GR-1+ splenocytes, here we also targeted those cells at the stage of immunization, prior to *T. cruzi* challenge. Notably, 5FU administration before each dose of TSf-ISPA vaccine was able to significantly ameliorate survival and decrease parasitemia levels of TSf-ISPA-vaccinated and infected mice. Overall, this work supports that targeting MDSCs may be a valuable tool during vaccine design against *T. cruzi*, and likely for other pathologies that are characterized by the subversion of the immune system.

## Introduction

The protozoan parasite *Trypanosoma cruzi* (*T. cruzi)* is the etiological agent of Chagas disease. More than 6 million people are infected and 65-100 million people live in areas at risk of *T. cruzi* infection worldwide (Lidani et al., 2019). In addition, there are 8000 to 15000 annual cases of vertical transmission (Picado et al., 2018) and 50000 annual deaths (Lidani et al., 2019).

Since the available treatment options for chronic patients have limited efficacy and present significant side effects (Sales Junior et al., 2017), Chagas disease remains a serious health problem not only in Latin America but also in non-endemic countries as a consequence of increasing international migration of infected individuals (Pérez-Molina and Molina, 2018). In addition, although notable progress has been made in vaccinology during the last decades (Koff et al., 2013; Amanna and Slifka, 2018), *T. cruzi* is one of the pathogens for which a vaccine is still not available. One striking characteristic shared among several microorganisms of this group is the ability to subvert the host immune system (Koff et al., 2013; Eberhardt and Barry, 2014; Flávia Nardy et al., 2015; Cardillo et al., 2015; Cabrera and Marcipar, 2019). In the particular case of *T. cruzi*, it has been shown that it possesses several strategies to generate an immunosuppressed state during acute experimental infection (Tarleton, 1988; Abrahamsohn and Coffman, 1995; DosReis et al., 1995; Michelin et al., 2005; Flávia Nardy et al., 2015; Cardoso et al., 2016; Morrot et al., 2016). Cells like CD11b+ GR-1+ myeloid-derived suppressor cells (MDSCs) (Goñi et al., 2002; Cuervo et al., 2011; Arocena et al., 2014; Fresno and Gironès, 2018), CD4+ Foxp3+ regulatory T cells (Tregs) (Mariano et al., 2008; Araújo et al., 2011; González et al., 2015; Prochetto et al., 2017; Araujo Furlan et al., 2018; Cabrera and Marcipar, 2019), regulatory dendritic cells (Van Overtvelt et al., 1999; Poncini et al., 2008; Poncini et al., 2015; Poncini and González-Cappa, 2017), γδ T cells (Cardillo et al., 2015), and suppressive IL-10-producing neutrophils (Tosello Boari et al., 2012) have been mentioned among the cellular components with regulatory/suppressor capacity that may play a role during the infection process.

We recently reviewed the studies that addressed the involvement of MDSCs and Tregs during acute experimental *T. cruzi* infection (Cabrera and Marcipar, 2019). Currently, MDSCs are considered a heterogeneous population of myeloid origin and immature state that has remarkable ability to suppress T-cell responses (Gabrilovich and Nagaraj, 2009). In mice, these cells are generally defined by the expression of both CD11b and Gr-1 markers with suppressor capacity, and two subpopulations are generally identified: G-MDSCs (CD11b+ Ly6G+ Ly6C^low/+^, also called granulocytic (G-MDSCs), and M-MDSCs (CD11b+ Ly6G^-^ Ly6C+, also called monocytic-MDSCs) (Gabrilovich and Nagaraj, 2009). Several molecules and mechanisms have been associated with suppression by MDSCs, including at least arginase, nitric oxide (NO), reactive oxygen species (ROS), transforming growth factor-β (TGF-β), and interleukin-10 (IL-10) (Condamine and Gabrilovich, 2011).

To date, reports addressing MDSCs indicate that this population plays a notable role during acute *T. cruzi* infection. For instance, the percentage of CD11b+ GR-1+ (Ly6G+/Ly6C+) cells in total spleen cells may increase from 2-5% to nearly 20-30% in acutely infected mice (Goñi et al., 2002; Arocena et al., 2014; Prochetto et al., 2017; Poncini and González-Cappa, 2017). Moreover, it has been shown that depletion of MDSCs using 5-fluorouracil (5FU) at day 10 or 15 of post-infection (p.i.) reduced mice survival to nearly 0% at day 21 p.i., strongly supporting that MDSCs plays a critical role in the control of inflammation, as previously suggested (Arocena et al., 2014).

Notably, despite ample evidence supporting that the regulatory/suppressor arm of the immune system is involved during the acute phase of *T. cruzi* infection, almost no vaccine study has addressed the utility to target the regulatory/suppressor axis in order to improve the vaccine efficacy against this parasite (Prochetto et al., 2017). Similarly, no studies have explored if MDSCs affect the immune response during the assessment of vaccine candidates against *T. cruzi*. This approach has special interest, since MDSCs may also decrease the expected effector response that should be elicited by any vaccine candidate. We reported that immunization with a trans-sialidase-based vaccine (TSf-ISPA) was able to decrease spleen MDSCs (mainly G-MDSCs) while the number of Tregs in the spleen was increased, a result that correlated with a significant increase of mouse survival against *T. cruzi* infection (Prochetto et al., 2017). These data are in line with studies performed in other infection models also showing improved protection with the use of vaccines associated with decreased levels of MDSCs after pathogen challenge (Heithoff et al., 2001; Sui et al., 2014). In addition, in the field of cancer study, ongoing clinical trials attempt to improve vaccine efficacy by targeting MDSCs (Fleming et al., 2018; Liu et al., 2018).

Thus, according to the data that show the importance of immunosuppression and MDSCs during *T. cruzi* infection, the aim of this work was to analyze whether MDSCs could modulate the immune response even in the context of the TSf-ISPA vaccine and assess the potential use of MDSCs as an additional target to improve rational vaccine design against *T. cruzi*.

## Material and Methods

### Mice

BALB/c female mice (6-8 weeks old) used in all experimental procedures were obtained from the Centro de Medicina Comparada, Instituto de Ciencias Veterinarias del Litoral (ICIVET-CONICET) Universidad Nacional del Litoral (UNL), Argentina. All protocols for animal studies were approved by the Animal Care & Use Committee of the Facultad de Bioquímica y Ciencias Biológicas, UNL, according to the Institutional guidelines that are based on international ethical guidelines for biomedical research involving animals.

### Immunization Schedules, Infection Protocol, and 5FU Treatment

BALB/c mice (n =5-6/group) were immunized with three subcutaneous doses, one every two weeks, containing 10 μg of a fraction of the trans-sialidase protein (TSf) (Prochetto et al., 2017) with 3 ul of ISPA as adjuvant. ISPA adjuvant is composed of liposomes with cage like structures of 73,0 ± 1,5 nm as assessed by dynamic light scattering. The components of the particles are phosphatidylcholine (DPPC), cholesterol (CHOL), sterylamine (STEA), tocopherol (TOCOP) and saponin, as previously described (Bertona et al., 2017).

Control groups were immunized with phosphate buffered saline (PBS) solution, following the same protocol. For infection, mice were challenged intraperitoneally with 900 or 1500 bloodstream trypomastigotes of Tulahuen strain, as indicated, 15 days after the last immunization. Parasites employed belong to the discrete typing unit VI (DTU VI) and were maintained by serial passages in Cbi suckling mice. Cbi mice were bred in the animal facilities of School of Medicine of Rosario, following protocols for animal studies approved by the Institutional Bioethics and Biosecurity Committees (Resolution N 3913/2008). According to the experiment, mice were treated intraperitoneally with 5FU (Sigma Aldrich) 50mg/kg, a dose previously described in a similar model of infection (Arocena et al., 2014). 5FU treatment was administered one day before vaccine (group TSf-ISPA Tc+ 5FU pre-vaccine); one day before infection together with a dose at day 5 p.i. (group TSf-ISPA Tc+ 5FU -1 and 5 p.i.), or at day 15 p.i. (group TSf-ISPA Tc+ 5FU).

Other authors and us have previously reported that 5FU administration with the dose employed did not affect significantly the numbers of B cells, T cells, or dendritic cells (DC) in treated mice (Vincent et al., 2010; Arocena et al., 2014; Poncini and González-Cappa, 2017; Khosravianfar et al., 2018). We also described that 5FU did not affect the parasite *T. cruzi* with the dose employed (Poncini and González-Cappa, 2017).

### Parasitemia and Survival

Parasitemia was monitored at day 20 p.i. by examining 5 μl of blood directly under microscope (Leica Microsystems), as previously described (Prochetto et al., 2017). Survival was recorded until day 21 of infection or until day 35 p.i., according to the experiment. Previously we showed that treatment with TSf alone or ISPA alone does not confer protection in terms of parasitemia and survival (Prochetto et al., 2017).

### Flow Cytometry and Cell Culture

Groups of mice were immunized with TSf-ISPA or PBS and treated or not with 5FU as described above. At day 21 p.i., mice were sacrificed and spleens were aseptically harvested and homogenized. Absolute numbers of spleen were determined by counting cells in Neubauer chamber using Turk solution and then red blood cells were eliminated by lysis with a solution of NH_4_Cl. Then, splenocytes were re-suspended in PBS with 3% fetal bovine serum (Gibco) and 0,1% azide for direct flow cytometry, or in RPMI1640 medium (Gibco) supplemented with 10% fetal bovine serum, 2% penicillin (100 μg/mL) and streptomycin (100 U/mL) (Gibco) and 0.4 mM 2-mercaptoethanol for *in vitro* culture.

For flow cytometry, 1×10^6^ cells were first incubated with anti-FcγIII/II receptor antibody and then stained with appropriate combinations of the following antibodies from BD-Pharmingen: anti-CD11b, anti-Ly6G, anti-Ly6C, anti-CD4, anti-CD11c, anti-CD80, anti-CD40, anti-MHCII, and anti-CD8α according to the manufacturer´s instruction in appropriate combinations of fluorophores for posterior analysis. The MFI index of CD40 in CD11c^high^ dendritic cells was calculated by dividing the MFI of CD40 in each group of mice/MFI of CD40 in non-infected mice. A similar formula was used to calculate the MFI index for CD80 and MHCII.

Intranuclear staining with anti-Foxp3 from Miltenyi Biotec was used following the manufacturer´s instruction.

For *in vitro* culture, 1×10^6^ cell/mL/well were cultured in 24 or 48-well plates (Nunc) in supplemented RPMI. Splenocytes were stimulated with concanavaline A (ConA) (Sigma-Aldrich) (5ug/ml) and *T. cruzi* homogenate or RPMI as control. After 72 hours at 37°C and 5% CO_2_, cells were harvested, incubated with anti-FcγIII/II receptor antibody and then stained with anti-CD8α, anti-CD107a and anti-CD44 in appropriate combinations of fluorophores for posterior analysis (**Supplementary Table I** shows the panel of antibodies, fluorochromes and combinations used).

For Ki-67 assay, splenocytes were cultured as previously described, and after CD4 surface staining, intranuclear staining of Ki-67 cells was performed using the Ki-67 antibody and the staining kit from BD-Pharmingen. The proliferation ratio was measured by dividing the percentage of Ki-67+ in CD4+ cells in each group/Ki-67+ in CD4+ cells in non-infected mice.

Samples were acquired on an Attune NxT cytometer (Invitrogen, Thermofisher) and analyses were performed using FlowJo software.

For MTT assay, splenocytes (1×10^6^ cell/mL/well) from each experimental group (non-infected; PBS Tc+; TSf-ISPA Tc+ and TSf-ISPA Tc+ 5FU) were cultured in 24 or 48-well plates (Nunc) in supplemented RPMI. Cells were stimulated with ConA (5ug/ml) and *T. cruzi* homogenate or RPMI as control. In addition, aminoguanidine 0.5 mM, a well-known iNOS inhibitor, was added to some wells as indicated in the Figures. After 72 hours 0.5 mg/ml MTT (Invitrogen, Thermofisher) was added to each well. After 4 additional hours, culture media were removed and 100 ul DMSO was added to each well. Finally, optical density (OD) was measured as previously reported (Mosmann, 1983). Relative increase in proliferation was measured by dividing OD of each group treated with aminoguanidine by the OD of the same group without aminoguanidine treatment.

### IFN-γ Determination From Plasma and *In Vitro* Culture

Groups of mice were immunized with TSf-ISPA or PBS and treated or not with 5FU as described above. At day 21 p.i., plasma samples were collected and IFN-γ, IL-10 and GM-CSF plasma levels were measured using ELISA kits from BD-biosciences, according to manufacturer´s instruction.

In addition, the levels of IFN-γ and IL-10 were measured in supernatants from cell cultures.

### Statistical Analyses

Multiple groups were first analyzed using non-parametric tests: Kruskall-Wallis test for k samples and then Mann-whitney test was employed to analyze differences between two particular groups. Mantel-Cox Long rank test was used to evaluate survival curves. All analyses were performed using GraphPad Prisma 6.0 software (GraphPad, California, USA). Significance is indicated with (*) when p<0.05 between the indicated groups.

## Results

### TSf-ISPA Immunization Allows MDSC Depletion Using 5FU in the Acute Phase of *T. cruzi* Infection

**Figure 1:**
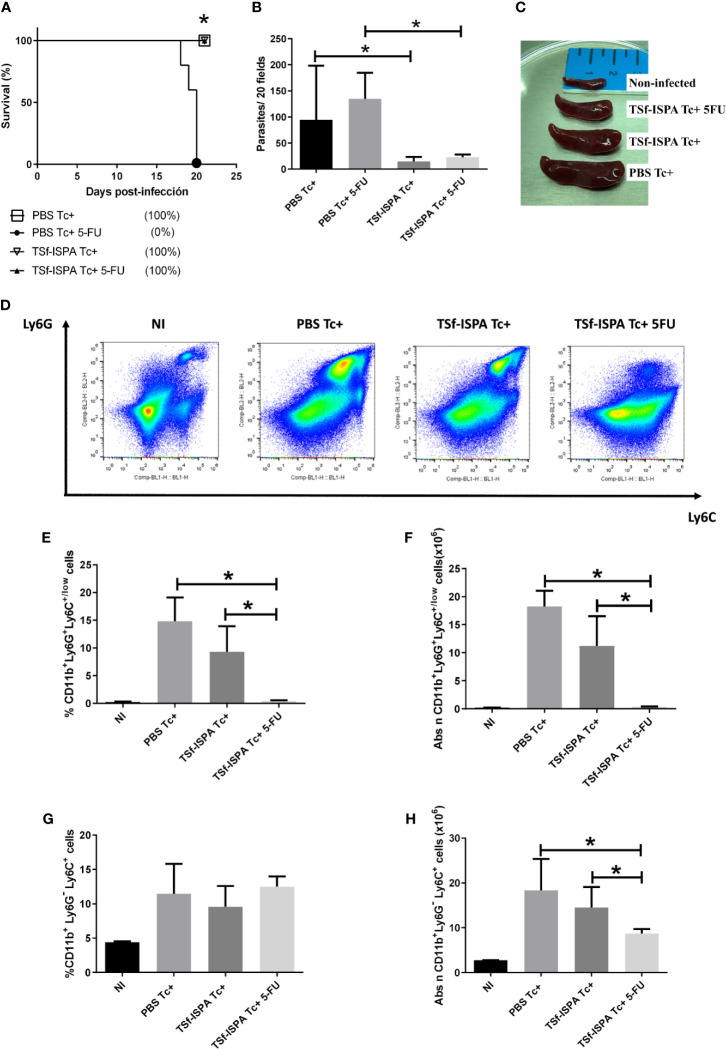
5FU treatment at day 15 p.i. strongly affects MDSCs and clinical parameters of *T. cruzi* infection. Mice immunized with TSf-ISPA or PBS were treated or not with 5FU and challenged with 900 trypomastigotes of the Tulahuen strain. **(A)** Survival rates are shown for TSf-ISPA immunized and infected mice (TSf-ISPA Tc+), PBS-inoculated and infected mice (PBS Tc+), TSf-ISPA immunized, infected and 5FU-treated mice (TSf-ISPA Tc+ 5FU) and PBS-inoculated, infected and 5FU-treated mice (PBS Tc+ 5FU). **(B)** Parasitemia from PBS Tc+ mice, PBS Tc+ 5FU mice, TSf-ISPA Tc+ mice and TSf-ISPA Tc+ 5FU at day 20 p.i. At 21 day p.i., mice were sacrificed to analyze the efficacy of MDSC depletion and the expression of Ly6C, Ly6G and CD11b were assessed by flow cytometry (FACS) in control non-infected (NI), PBS Tc+, TSf-ISPA Tc+ and TSf-ISPA Tc+ 5FU. **(C)** Representative picture of the size of the spleens from experimental groups of mice at day 21 p.i. (Mice from the PBS Tc+ 5FU group died before day 21 p.i. when spleens were extracted). **(D)** Representative dot plots of the expression of Ly6G and Ly6C in mouse spleen from each experimental group. **(E)** Percentage of CD11b+ Ly6G+ Ly6C^+/low^ cells. **(F)** Absolute number of CD11b+ Ly6G+ Ly6C^+/low^ cells (x10^6^), **(G)** Percentage of CD11b+ Ly6G- Ly6C+ cells. **(H)** Absolute number of CD11b+ Ly6G- Ly6C+ cells (x10^6^). Data are expressed as means + standard deviations. The results are representative of two independent experiments (n=5 mice per group), *p < 0,05, Mantel–Cox Long rank test was used for survival analysis between all groups *vs* PBS Tc+ 5FU in Figure **(A)** *p < 0,05, Mann-Whitney test was used for comparison between indicated groups in graphics **(B, E, F, H)**.

Numerous studies have used the model of BALB/c mice infection by *T. cruzi* of Tulahuen strain. Notably, similar results have been obtained concerning the increase in parasitemia and mortality from day 15 to day 21 p.i. (Arocena et al., 2014; Bontempi et al., 2015; Prochetto et al., 2017; Bontempi et al., 2017; Stempin et al., 2017). According to these data and considering that we and others have reported that spleen MDSCs increases mainly after day 15 p.i. (Arocena et al., 2014; Prochetto et al., 2017), we sought to analyze the *in vivo* influence of MDSCs on the immune response at the moment of their significantly highest increase. For this purpose, 5-fluorouracil (5FU) was used to selectively deplete MDSCs, as previously shown in several models, including models of *T. cruzi* infection (Arocena et al., 2014; Poncini and González-Cappa, 2017). Since we have previously described that TSf-ISPA immunization increased mouse survival, notably decreasing G-MDSCs (Prochetto et al., 2017), we speculated that TSf-ISPA immunization would allow mice to tolerate an almost complete depletion of MDSCs during the acute *T. cruzi* infection. In addition, a slightly lower dose of 900 Tulahuen trypomastigotes was selected for these experiments in an attempt to maintain some level of survival of the control group. Interestingly, despite the lower infective dose employed, 100% of PBS-treated and infected mice that received 5FU (PBS Tc+ 5FU) succumbed to the parasite challenge before day 21 p.i., likely as a consequence of an exacerbated immune response, as previously suggested (Arocena et al., 2014). In contrast, 60-100% of immunized and 5FU-treated mice (TSf-ISPA Tc+ 5FU) survived to the challenge at day 21 p.i. (**Figure 1A** and **Supplementary Table II**). Concerning the groups of mice that did not received 5FU, 60-100% of PBS-treated mice (PBS Tc+) survived to the parasite challenge, whereas 100% of immunized mice (TSf-ISPA Tc+) were alive at day 21 p.i. (**Figure 1A** and **Supplementary Table II**). Parasitemia assessment showed that PBS Tc+ mice had more parasites than TSf-ISPA Tc+ mice, whereas PBS Tc+ 5FU mice had more parasites than TSf-ISPA Tc+ 5FU mice (**Figure 1B** and **Supplementary Table II**).

At day 21 p.i., mice were sacrificed to check splenomegaly and MDSC depletion. A progressive increase of spleen size was observed as follow: non-infected mice (NI)<TSf-ISPA Tc+ 5FU mice<TSf-ISPA Tc+ mice< PBS Tc+ mice (**Figure 1C**). In strong correlation with spleen size, flow cytometry showed that the percentage and absolute number of G-MDSCs (CD11b+ Ly6G+ Ly6C^+/low^ cells) increased in a manner that correlated with the spleen size of the groups analyzed: NI<=TSf-ISPA Tc+ 5FU<TSf-ISPA Tc+< PBS Tc+ mice (**Figures 1E, F**). **Figure 1D** shows representative dot plots of Ly6C and Ly6G expression in all experimental groups. On the other hand, the percentage of M-MDSC cells (CD11b+ Ly6G- Ly6C+) was similar in all groups of infected mice, whereas the absolute number changed with the same trend as G-MDSCs: NI<TSf-ISPA Tc+ 5FU<TSf-ISPA Tc+< PBS Tc+ mice (**Figures 1G, H**) (**Supplementary Figure 1** shows the gating strategy).

Taken together, these results show that PBS-treated mice do not tolerate a challenge with 900 Tulahuen *T. cruzi* parasites if MDSCs are depleted by 5FU treatment at day 15 p.i. In contrast, a proportion of TSf-ISPA-immunized and infected mice tolerated and survived infection allowing the *in vivo* study of the influence of MDSCs on several components of the effector and regulatory immune response during the acute phase of *T. cruzi* infection.

### iNOS Expression and Immunosuppressive Capacity of MDSCs

It has been shown that CD11b+ GR-1+ cells from *T. cruzi* infected mice expressed iNOS and were able to inhibit T cell proliferation (Goñi et al., 2002; Arocena et al., 2014).

Although the suppressive capacity of MDSCs and iNOS expression have already been demonstrated in similar models of *T. cruzi* infection (Goñi et al., 2002; Arocena et al., 2014), we also assessed the suppressive capacity (**Supplementary Figure 2**) and iNOS expression to confirm the similarity of our model with Tulahuen *T. cruzi* infection to those previously reported.

**Figure 2:**
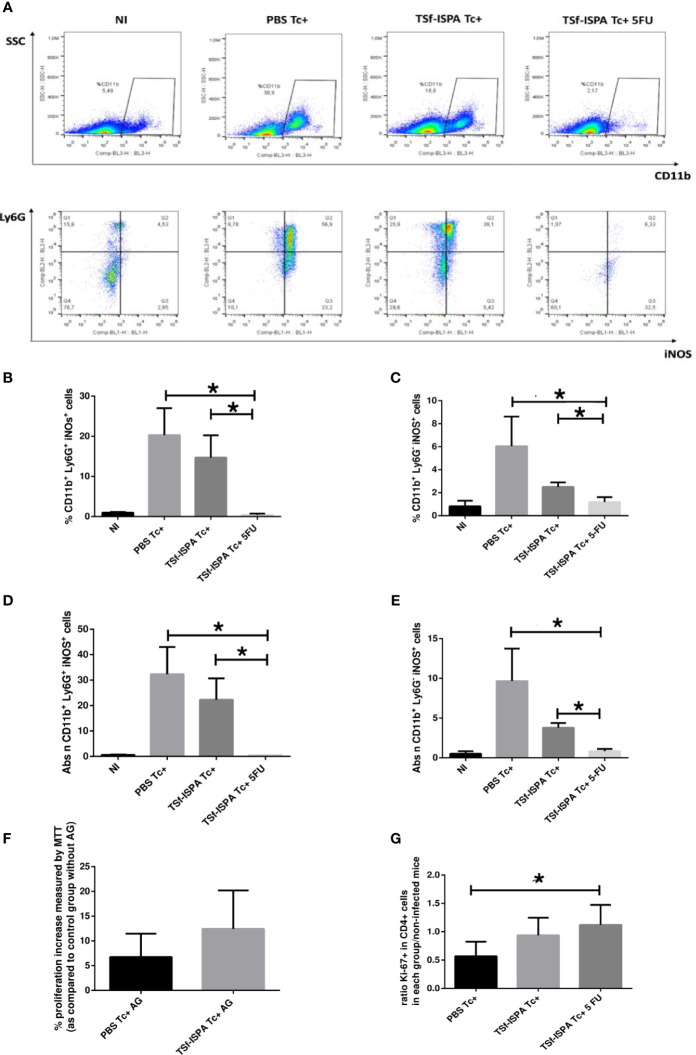
iNOS expression by MDSC splenocytes. Mice immunized with TSf-ISPA or PBS were treated or not with 5FU and challenged with 900 trypomastigotes of the Tulahuen strain. At 21 days p.i., mice were sacrificed to analyze the expression of iNOS in CD11b+ Ly6G+ (G-MDSCs) and CD11b+ Ly6G- (which include M-MDSCs) cells by FACS in control non-infected (NI), PBS-inoculated and infected mice (PBS Tc+), TSf-ISPA immunized and infected mice (TSf-ISPA Tc+) and TSf-ISPA immunized, infected and 5FU treated mice (TSf-ISPA Tc+ 5FU). **(A)** Representative dot plots of the expression of CD11b *vs* side scatter and of iNOS *vs* Ly6G expression in the region indicated. **(B)** Percentage of CD11b+ Ly6G+ iNOS+ cells. **(C)** Percentage of CD11b+ Ly6G- iNOS+ cells, **(D)** Absolute number of CD11b+ Ly6G+ iNOS+ cells (x10^6^). **(E)** Absolute number of CD11b+ Ly6G- iNOS+ cells (x10^6^). **(F)** Splenocytes from NI, PBS Tc+ and TSf-ISPA Tc+ mice were cultured and stimulated with ConA, *T. cruzi* homogenate and aminoguanidine (AG), or with ConA, *T. cruzi* homogenate and without AG, an iNOS inhibitor. After 72 hs, MTT was used to measure proliferation increase using the formula explained in materials and methods. **(G)** Splenocytes from each experimental group were cultured and stimulated with conA and *T. cruzi* homogenate. After 72 hs, proliferation was measured by FACS using the Ki-67 proliferation marker and the formula detailed in materials and methods. Data are expressed as means + standard deviations. Results are representative of two independent experiments (n = 3-5 mice per group, according to the survival of the experiment) *p < 0,05 Mann-Whitney test was used for comparison between indicated groups.

As expected, PBS Tc+ mice showed a notable increase in the percentage and absolute number of spleen G-MDSCs cells expressing iNOS (CD11b+ Ly6G+ iNOS+) as compared to non-infected mice. In addition, TSf-ISPA Tc+ mice showed lower levels of spleen G-MDSCs expressing iNOS than PBS Tc+ mice, a result that correlates with the decrease of G-MDSCs observed in TSf-ISPA mice, as previously reported (Prochetto et al., 2017). Finally, depletion of G-MDSCs in TSf-ISPA Tc+ 5FU mice caused a marked decrease in the percentage and absolute number of spleen G-MDSCs expressing iNOS that almost reached the basal levels of non-infected mice (**Figures 2A, B, D**). In addition, similar changes were observed concerning the cells that include the M-MDC population (CD11b+ Ly6G- iNOS+), as shown in **Figures 2A, C, E**. Additionally, the functional relevance of iNOS in the suppression capacity of MDSCs was also assessed. For this purpose, splenocytes from each experimental group were ConA stimulated *in vitro* in the presence of aminoguanidine (AG), a selective iNOS inhibitor. **Figure 2F** shows that addition of AG to the cultures caused an increase in the proliferation of the splenocytes from both PBS Tc+ and TSf-ISPA Tc+ mice, as measured by a standard MTT assay.

Results presented herein show that both G-MDSC and cells that include M-MDSC splenocytes from *T. cruzi* infected mice express iNOS, which is a feature of MDSC cells and has been involved as an important mechanism of suppression by these cells during acute *T. cruzi* infection. In addition, the depletion of MDSCs almost overlapped with iNOS+ expressing cells reduction, strongly suggesting that MDSCs largely accounts for the increase of iNOS+ splenocytes after *T. cruzi* infection. Moreover, the addition of AG to splenocytes cultured with ConA increased proliferation, which strongly supports the immunosuppressor role of MDSC iNOS+ cells in our experimental condition. Additionally, splenocytes from TSf-ISPA Tc+ and TSf-ISPA Tc+ 5FU mice, (which have lower and almost complete depletion of MDSCs, respectively) showed higher expression of Ki-67 in CD4+ cells than splenocytes from PBS Tc+ mice, after *in vitro* culture with ConA (**Figure 2G**), showing that lower levels of MDSCs allow higher CD4+ cell proliferation.

### MDSC Depletion Notably Potentiates the CD8 Effector Response Elicited by TSf-ISPA Formulation

It was reported that 5FU treatment not only causes a marked increase of mortality of *T. cruzi*-infected mice, but also has an important influence on the effector immune response, increasing the number of CD8+ CD107a+ spleen cells and the level of plasma cytokines, such as IFN-γ, IL-6 and TNF-α (Arocena et al., 2014). Since we have previously shown that TSf-ISPA formulation increased several immune parameters related to the effector immune response (Prochetto et al., 2017), we sought to evaluate whether MDSC depletion could potentiate the effector response elicited by the TSf-ISPA vaccine.

**Figure 3:**
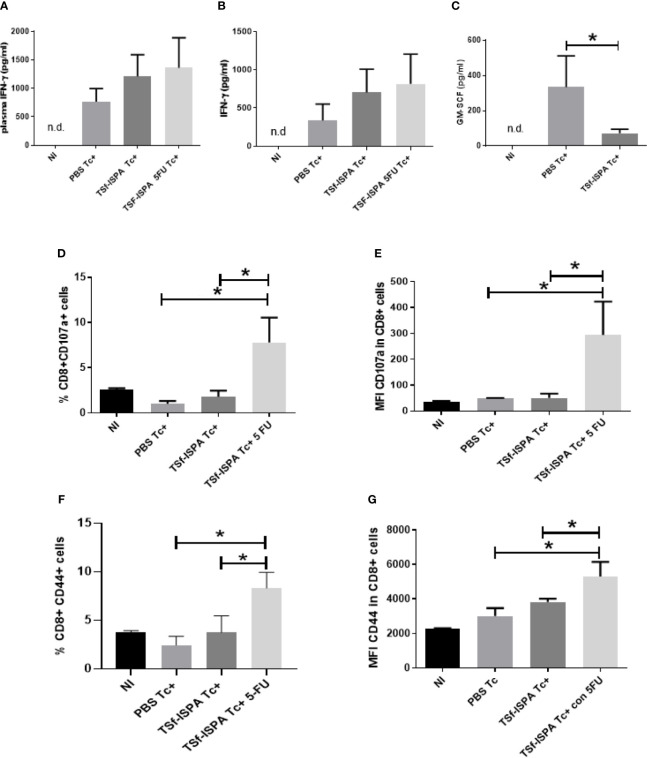
MDSC depletion notably potentiates the CD8 effector response elicited by TSf-ISPA formulation. Mice immunized with TSf-ISPA or PBS were treated or not with 5FU and challenged with 900 trypomastigotes of the Tulahuen strain. **(A)** At 21 day p.i. plasma was obtained and the levels of IFN-γ was measured by ELISA in control non-infected (NI), PBS-inoculated and infected mice (PBS Tc+), TSf-ISPA immunized and infected mice (TSf-ISPA Tc+) and TSf-ISPA immunized, infected and 5FU treated mice (TSf-ISPA Tc+ 5FU). **(B)** Mice were sacrificed at day 21 p.i. and splenocytes were cultured with conA and *T. cruzi* homogenate for analyzing the levels of IFN-γ by ELISA in culture supernantant of each experimental group. **(C)** Plasma obtained at 21 p.i. was used to measure GM-CSF by ELISA in NI, PBS Tc+ and TSf-ISPA Tc+ mice. Finally, splenocytes cultured for 72 h were harvested and the expression of CD8, CD107a and CD44 were analyzed by FACS. **(D)** Percentage of CD8+ CD107a+ cells. **(E)** MFI of CD107a in CD8+ cells. **(F)** Percentage of CD8+ CD44+ cells, **(G)** MFI of CD44 in CD8+ cells. Data are expressed as means + standard deviations. Results are representative of two independent experiments (n = 3-5 mice per group, according to the survival of the experiment).*p < 0,05 between indicated groups of mice.

In the setting of our experimental model, the level of plasma IFN-γ at day 21 p.i. increased progressively in the following trend: NI<PBS Tc+ mice<TSf-ISPA Tc+ mice<TSf-ISPA Tc+ 5FU mice (**Figure 3A**). The same trend of IFN-γ production was observed in culture supernatants of splenocytes stimulated with ConA and *T. cruzi* homogenate, as shown in **Figure 3B**.

It has been reported that MDSC increases is dependent on IFN-γ during *T. cruzi* infection, since no increases in that population was observed in IFN-γ KO mice (Goñi et al., 2002). Interestingly, TSf-ISPA immunized mice showed higher levels of IFN-γ, but lower levels of spleen MDSCs than PBS-treated and infected mice. The trends found in plasma and in culture supernatants in these results are in line with our previous report showing that IFN-γ production is significantly increased in CD8+ T cells in vaccinated and infected mice (Prochetto et al., 2017). To explain this observation, we measured the levels of plasma GM-CSF, since this growth factor has been reported to be involved in *T. cruzi* infection (Olivares Fontt et al., 1996) and in the induction of MDSCs (Gabrilovich and Nagaraj, 2009). GM-CSF levels were found to be lower in TSf-ISPA Tc+ mice than PBS Tc+ mice (**Figure 3C**), suggesting that through the induction of GM-CSF *T. cruzi* is able to take advantage of the INF-γ production to induce a high increase of MDSCs. In contrast, TSf-ISPA-vaccinated mice seems to avoid the high increase of plasma GM-CSF allowing a higher increase of IFN-γ, which does not result in a correlated increase of MDSCs. The possibility that other cytokines or mediators also influence MDSCs increases during T*. cruzi* infection cannot be discarded. In addition, after *in vitro* culture, an increase in the percentage of CD8+ CD107a+ splenocytes was recorded in the TSf-ISPA Tc+ 5FU treated group (**Figure 3D**), as compared with all the other experimental groups. A similar result was observed regarding the MFI of CD107a in CD8+ (**Figure 3E**). In the same line, an increase of CD8+ CD44+ splenocytes were recorded in the TSf-ISPA Tc+ 5FU group of mice with respect to all the other groups (**Figure 3F**). Finally, similar results were observed regarding the MFI of CD44 in CD8+ cells (**Figure 3G**) (**Supplementary Figure 3** shows the gating strategy used). These data strongly suggest that the decrease of immunosuppressive MDSCs caused by 5FU treatment allows an enhanced effector response in TSf-ISPA vaccinated mice.

### MDSC Depletion Increases CD4+ Foxp3+ (Tregs)

**Figure 4:**
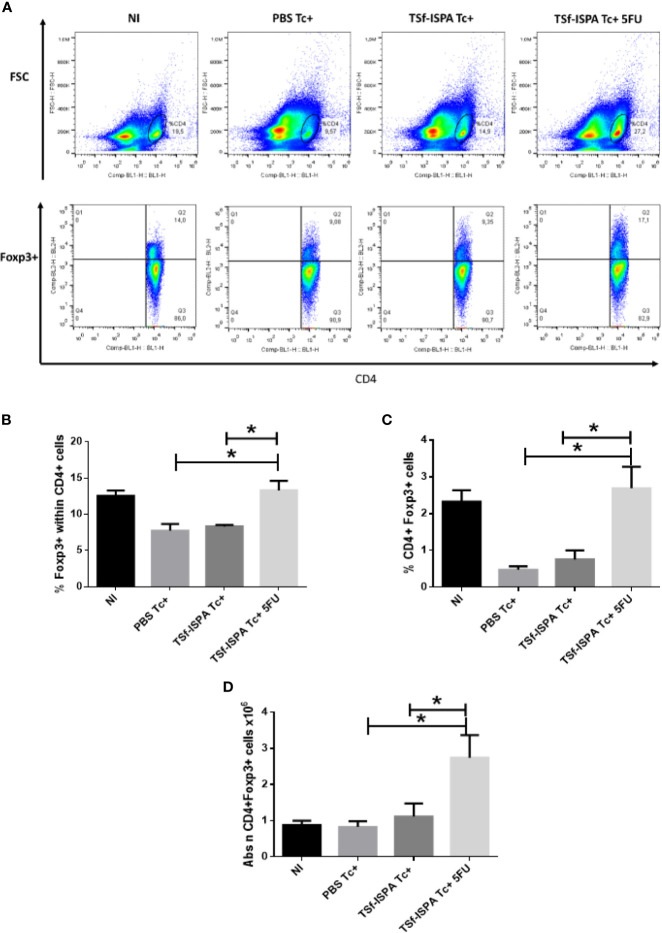
MDSC depletion increases Tregs. Mice immunized with TSf-ISPA or PBS were treated or not with 5FU and challenged with 900 trypomastigotes of the Tulahuen strain. At 21 day p.i., mice were sacrificed to analyze the expression of Foxp3 and CD4 by FACS in control non-infected (NI), PBS-inoculated and infected mice (PBS Tc+), TSf-ISPA immunized and infected mice (TSf-ISPA Tc+) and TSf-ISPA immunized, infected and 5FU treated mice (TSf-ISPA Tc+ 5FU). **(A)** Representative dot plots of the expression of CD4 *vs* forward scatter and CD4 *vs* Foxp3 expression in the indicated region of experimental groups under study: **(B)** Percentage of Foxp3+ expression in CD4+ cells. **(C)** Percentage of CD4+ Foxp3+ cells, **(D)** Absolute number of CD4+ Foxp3+ cells x10^6^. Data are expressed as means + standard deviations. The results are representative of three independent experiments (n = 3-5 mice per group, according to the survival of the experiment). *p < 0,05 between indicated groups of mice.

We have previously shown that TSf-ISPA vaccination not only induces a decrease of CD11b+ GR-1+ spleen cells after *T. cruzi* infection, but also causes a simultaneous increase in the number of spleen Tregs. According to our previous result, we speculated that if a decrease of MDSCs correlated with an increase of Tregs, an almost complete depletion of MDSCs should cause a higher increment of Tregs. In line with this hypothesis, TSf-ISPA Tc+ 5FU mice (which were depleted of MDSCs) showed a marked increase in Foxp3+ expression in CD4+ cells and in the percentage of Tregs, compared to PBS Tc+ and TSf-ISPA Tc+ mice (**Figures 4A–C**). In addition, an increase in the absolute number of Tregs was observed in the spleen of TSf-ISPA Tc+ 5FU group with respect to all other experimental groups (**Figure 4D**).

Thus, our results show that the depletion of MDSCs in TSf-ISPA immunized and *T. cruzi* infected mice results in a striking increase of spleen Tregs.

### G-MDSC Depletion Influences the Spleen Dendritic Cell Population

Since DCs are key players in the immune system, influencing both immunity and tolerance, we sought to evaluate whether TSf-ISPA immunization and depletion of MDSCs may also affect DCs.

We first assessed whether TSf-ISPA immunization and 5FU-treatment would influence maturation/activation markers in CD11c^high^ spleen DCs. For that purpose, the expression of CD80, CD40 and MHCII was measured and relativized against the NI mice, as detailed in material and methods.

**Figure 5:**
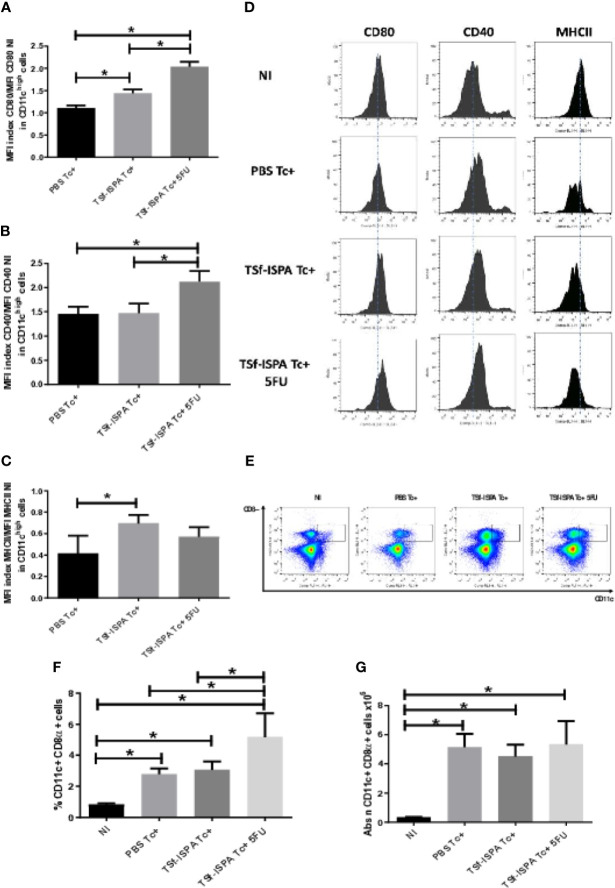
MDSC depletion and dendritic cells. Mice immunized with TSf-ISPA or PBS were treated or not with 5FU and challenged with 900 trypomastigotes of the Tulahuen strain. At day 21 p.i., mice were sacrificed to analyze the expression of CD11c, CD80, CD40, MHCII and CD8α by FACS in control non-infected (NI), PBS-inoculated and infected mice (PBS Tc+), TSf-ISPA immunized and infected mice (TSf-ISPA Tc+) and TSf-ISPA immunized, infected and 5FU treated mice (TSf-ISPA Tc+ 5FU). **(A)** Index of MFI CD80/MFI CD80 of non-infected mice in CD11c^high^ cells. **(B)** Index of MFI CD40/MFI CD40 of non-infected mice in CD11c^high^ cells. **(C)** Index of MFI MHCII/MFI MHCII of non-infected mice in CD11c^high^ cells. **(D)** Representative histograms of the expression of CD80, CD40 and MHCII in CD11c^high^ cells in the experimental groups of mice under study: **(E)** Representative dot plots of the expression of CD11c and CD8 in the experimental groups of mice. **(F)** Percentage of CD11c+ CD8α+ DCs. **(G)** Absolute number of CD11c+ CD8α+ cells in the spleen x10^6^. Data are expressed as means + standard deviations. The results are representative of two independent experiments (n = 3-5 mice per group, according to the survival of the experiment). *p < 0,05 between indicated groups of mice.

**Figures 5A**, **D** shows that the MFI of CD80 in CD11c^high^ DCs from infected mice increased in the following order: PBS Tc+<TSf-ISPA Tc+<TSf-ISPA Tc+ 5FU, suggesting that TSf-ISPA immunization increases CD80 expression in CD11c^high^ DCs, compared to PBS Tc+. In addition, 5FU-treatment increased CD80 expression in CD11c^high^ DCs even more than immunization alone.

Moreover, MFI of CD40 in CD11c^high^ DCs increased significantly only in TSf-ISPA Tc+ 5FU as compared to PBS Tc+ and TSf-ISPA Tc+ mice (**Figures 5B**, **D**), whereas the MFI of MHCII in CD11c^high^ DCs increased significantly in TSf-ISPA Tc+ mice as compared to PBS Tc+ mice (**Figures 5C**, **D**). In the case of MHCII expression, it is noteworthy that in all infected groups the index is lower than 1, showing that the MHCII expression is decreased with respect to non-infected mice (**Supplementary Figure 4** shows the gating strategy).

It was described that the CD8α+ subtype of CD11c conventional DCs, expressing a dimer of CD8α, are particularly suited for eliciting a response of T CD8 cells that express a dimer CD8αβ (Heath et al., 2004; Merad et al., 2013). Since the CD8 response is critical to cope with *T. cruzi* infection (Martin et al., 2006; Acosta Rodríguez et al., 2019), the influence of MDSC depletion on the CD8α+ subtype of DCs was assessed. Regarding the absolute numbers and according to the splenomegaly, all groups of infected mice showed an increase in the absolute number of CD11c+ CD8α+ DCs as compared to non-infected mice (**Figures 5E**, **G**). In addition, PBS Tc+ and TSf-ISPA Tc+ mice showed an increase in the percentage of CD11c+ CD8α+ DCs in the spleen as compared to non-infected mice, and TSf-ISPA Tc+ 5-FU mice showed a higher increase in the percentage of those cells as compared to PBS Tc+ and TSf-ISPA Tc+ mice (**Figure 5F**) (**Supplementary Figure 4** shows the gating strategy).

### Effect of 5FU Treatment During the First Week of *T. cruzi* Infection

MDSC depletion of vaccinated mice at day 15 p.i. allowed us to study the alterations that occur in the percentage, absolute number and phenotype of several immune populations in the absence of MDSCs. However, this late depletion did not improve mouse survival, which might be related to an exacerbated inflammation, as previously suggested (Arocena et al., 2014). Thus, to assess whether MDSCs could be targeted by a different strategy that could cause a potential and beneficial effect on the outcome of infection, 5FU was administered one day before infection (-1) and at day 5 p.i. and mice were challenged with a higher dose (1500 parasites) to reduce the survival of TSf-ISPA-vaccinated mice to a useful range.

**Figure 6:**
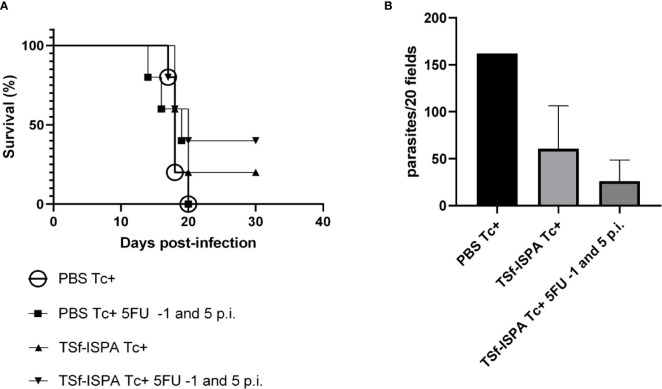
5FU treatment at day -1 and 5 p.i. and the protective capacity of TSf-ISPA vaccine. Mice immunized with TSf-ISPA or PBS were treated or not with 5FU at day -1 and 5 p.i. and challenged with 1500 trypomastigotes of the Tulahuen strain. **(A)** Survival rates are shown for PBS-inoculated and infected mice (PBS Tc+), PBS Tc+ treated with 5FU at day -1 and 5 p.i. (PBS Tc+ 5FU -1 and 5 p.i.), TSf-ISPA immunized and infected mice (TSf-ISPA Tc+) and TSf-ISPA Tc+ treated with 5FU at day -1 and 5 p.i (TSf-ISPA Tc+ 5FU -1 and 5 p.i.); **(B)** Parasitemia at day 20 p.i. of PBS Tc+, TSf-ISPA Tc+ and TSf-ISPA Tc+ 5FU at day -1 and 5 p.i. (PBS Tc+ 5FU -1 and 5 p.i. mice were dead at day 20 p.i.) Data are expressed as means + standard deviations. The results are representative of two independent experiments (n=5 mice per group).

**Figure 6A** shows that a higher dose of the virulent *T. cruzi* Tulahuen strain notably affected mouse survival. All PBS-treated mice as well as the PBS-treated mice that received 5FU at day -1 and 5 p.i. succumbed to the challenge before day 21 p.i. Survival of TSf-ISPA-vaccinated mice ranged between 20-40%, whereas that of TSf-ISPA mice that received 5FU at day -1 and 5 p.i ranged between 40-80% range. Thus, although late depletion of MDSCs caused a decrease in the survival of vaccinated mice, early MDSC depletion caused a slight increase of survival of TS-ISPA vaccinated mice against a lethal dose of *T. cruzi* Tulahuen. In line with this result, the parasitemia analysis showed that TSf-ISPA-vaccinated mice that received 5FU had the lowest parasitemia level at day 20 p.i (**Figure 6B**). Since mortality increased at the dose employed, more animals would be needed to achieve statistical significance in these experiments, which show a trend suggesting that MDSC depletion causes a beneficial effect on TSf-ISPA-vaccinated and infected mice.

Interestingly, early 5FU administration of PBS-treated mice did not improve survival nor parasitemia levels of that group of mice, suggesting that the vaccine is necessary to prime the immune system before infection, in order to allow a beneficial effect of MDSC depletion.

### Effect of MDSC Depletion Before TSf-ISPA Immunization

Although the present results are useful to support that MDSCs are able to modulate the immune response even in the context of a vaccine candidate, the depletion of those cells after infection is an approach that cannot be used in next steps of preclinical or clinical vaccine assessment. Thus, to conclude this study we sought to analyze the usefulness of depleting MDSCs at the stage of immunization. This approach is of particular interest, since we previously showed that TSf-ISPA vaccine caused a slight but significant increase of spleen CD11b+ Gr-1+ cells during the immunization process (Prochetto et al., 2017). For this purpose, a group of mice were treated with 5FU one day before each dose of the TSf-ISPA vaccine.

**Figure 7:**
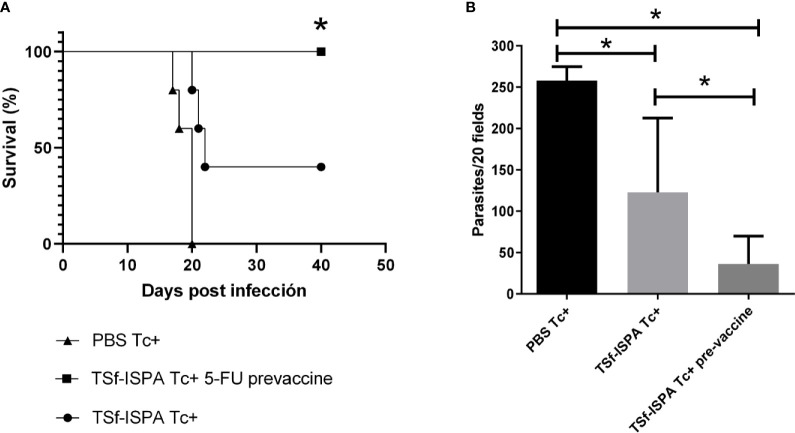
5FU treatment one day before each dose of TSf-ISPA vaccine. Mice immunized with TSf-ISPA or PBS were treated or not with 5FU one day before each dose of TSf-ISPA vaccine and then were challenged with 1500 trypomastigotes of the Tulahuen strain. **(A)** Survival rates are shown for PBS-inoculated and infected mice (PBS Tc+), TSf-ISPA immunized and infected mice (TSf-ISPA Tc+), and TSf-ISPA mice that received 5FU one day before each dose of the vaccine (TSf-ISPA Tc+ 5FU pre-vaccine), **(B)** Parasitemia at day 20 p.i. from PBS Tc+, TSf-ISPA Tc+, and TSf-ISPA Tc+ 5FU pre-vaccine. The results are representative of two independent experiments (n =5 mice per group. *p < 0,05 between indicated groups of mice.

Strikingly, after a lethal dose of 1500 parasites that caused 0% survival of PBS Tc+ mice and 20-40% survival of TSf-ISPA Tc+ mice (non 5FU-treated), an important percentage of survival, of between 80-100% was obtained for the TSf-ISPA Tc+ 5FU-treated (pre-vaccine) mice (**Figure 7A**). TSf-ISPA 5FU pre-vaccine mice showed decreased parasitemia levels as compared to TSf-ISPA Tc+ mice and PBS-treated and infected mice (PBS Tc+) at day 20 p.i. (**Figure 7B**). Taken together, these results strongly suggest that MDSCs could represent a relevant target to be considered during rational vaccine design against *T. cruzi*.

## Discussion

The aim of any vaccine candidate should include the elicitation of a proper immune response able to control the mechanisms used by pathogens to infect their hosts. This task could be difficult to achieve when pathogens are able to manipulate the immune response. Since it is well-known that *T. cruzi* is able to induce a marked immmunosuppressive state (Mariano et al., 2008; Arocena et al., 2014; Cardillo et al., 2015; González et al., 2015; Poncini et al., 2015; Prochetto et al., 2017), it is striking that very few vaccine studies have addressed simultaneously alterations of the effector and the regulatory/suppressor arm of the immune system during the assessment of a vaccine candidate against *T. cruzi*. Thus, given the lack of this kind of information, the aim of this work was to study whether MDSCs modulate the immune response against *T. cruzi*, even in the context of a vaccine with protective capacity, and the potential use of those cells as a vaccine target to enhance protection against *T. cruzi*.

In line with previous reports, MDSC depletion at day 15 p.i of PBS-treated mice resulted in severe mortality and 100% of depleted mice succumbed by day 21 p.i. with a dose of 900 parasites. Nonetheless, TSf-ISPA immunization allowed us to achieve 60-100% of mouse survival at day 21 p.i. after the almost complete depletion of G-MDSCs and partial depletion of M-MDSCs. In mice that were not treated with 5FU, spleen MDSCs expressed high levels of iNOS *in vivo*, a useful marker that has been described to be useful to distinguish MDSCs from neutrophils (Veglia et al., 2018). Noteworthy, after MDSC depletion, spleen iNOS expression was almost completely abolished, strongly supporting that MDSCs account for an important part of iNOS expression in the spleen. Although other studies showed and confirmed *in vitro* that spleen MDSCs from *T. cruzi*-infected mice have suppressive capacity related to iNOS (Goñi et al., 2002; Arocena et al., 2014), a broader study concerning the influence of MDSCs on other immune populations has scarcely been addressed, particularly, during the assessment of a vaccine candidate. The present results show that MDSC depletion notably reshaped the *in vivo* immune response of vaccinated and infected mice, influencing CD8 effector T cells, Tregs, and even the activation status of CD11c^high^ DCs.

Numerous reports, including vaccines studies, have highlighted the critical relevance of the CD8 response to control *T. cruzi* infection (Martin et al., 2006; Acosta Rodríguez et al., 2019). The present results show that MDSC depletion at day 15 p.i. highly increased the CD8 response in TSf-ISPA-vaccinated mice, even compared to vaccinated mice that were not treated with 5FU, strongly suggesting that MDSCs play an important role in suppressing CD8 cells, benefiting the parasite even in vaccinated mice. Remarkably, MDSC depletion resulted in an increased percentage and number of spleen Tregs. We have recently reviewed the reports addressing the role of Tregs during *T. cruzi* infection (Cabrera and Marcipar, 2019). Despite some discrepancies, a consensus may be formulated that Tregs play a limited or moderate role in increasing mouse survival, even if they restrain the CD8+ response and benefits parasite persistence. Taken together, the present results suggest that the marked increase of MDSCs plays a major biological role, even more notable than that of Tregs, since depletion of MDSCs results in a higher CD8 response in the context of an increased number of Tregs.

Although there is ample evidence of the mechanisms employed by MDSCs to suppress effector T cells, little is known about the potential interaction of MDSCs and Tregs. Since Foxp3 expression by CD4+ T cells correlates with suppressor activity irrespective of CD25 expression (Komatsu et al., 2009), first we addressed potential interactions between MDSCs and Tregs by studying alterations in the frequency and number of CD4+ Foxp3+ Tregs. According to the present results further research is in progress to study the mechanisms involved as well as the phenotype, specificity and suppressor capacity of the Tregs that increase after MDSC depletion. On the other hand, despite the role of Tregs during the acute phase of infection remains to be completely elucidated, most of the studies concerning the role of Tregs during the chronic phase support the beneficial role of these cells for the host (Araújo et al., 2011). In this sense, a vaccine that decreases parasitemia and increases Tregs may represent a valuable tool to control pathology, similarly to other vaccines that are already in use, such as BCG.

It has been described that *T. cruzi* is able to influence DC maturation, impairing the development of an efficient immune response (Van Overtvelt et al., 1999; Poncini et al., 2008; Poncini et al., 2015; Poncini and González-Cappa, 2017). Moreover, a recent study has proposed that MDSCs are able to kill DCs in a NO-dependent manner (Ribechini et al., 2019). In our experimental condition, where a high increase of MDSCs iNOS+ cells occur during acute *T. cruzi* infection, we observed that CD11c^high^ DCs from PBS-treated mice did not increase the expression of activation/maturation markers, like CD80, CD40 and MHCII, as compared to non-infected mice. In contrast, vaccinated mice (which have lower levels of MDSCs than PBS), as well as vaccinated and MDSC depleted mice, showed a significant increase in some of those activation markers, which correlated with protection capacity and the elicitation of an increased effector response.

An interesting point considering *T. cruzi* infection is the dual role attributed to several components of the immune system. For instance, it has been reported that DCs are a double-edged sword (Gil-Jaramillo et al., 2016), and in the particular case of monocyte-derived DCs, it has been described that those cells play a role in controlling parasite multiplication but also in interfering with the development of the immune response (Poncini and González-Cappa, 2017). Tregs could also play opposite roles during acute *T. cruzi* infection, increasing mouse survival but allowing parasite persistence (Cabrera and Marcipar, 2019). Reactive oxygen species (ROS), TGF-β and INF-γ may also play dual roles (Bahia-Oliveira et al., 1998; Hall and Pereira, 2000; Goñi et al., 2002; Ferreira et al., 2014; Goes et al., 2016; Paiva et al., 2018). INF-γ is a critical component of the Th1 profile, necessary to cope with infection, but this cytokine is also involved in immunosuppressive MDSCs induction during *T. cruzi* infection (Goñi et al., 2002). Thus, in a setting where the parasite subverts the immune system and forces its components to play dual roles, a vaccine candidate should be able to reestablish the subverted mechanisms, allowing a proper effector response. In our model, a better immune response would require the increase of IFN-γ levels but avoiding its dual role of increasing MDSCs. According to the “two-signal” model of MDSCs accumulation and activation (Condamine and Gabrilovich, 2011), an increase of immunosuppressive MDSCs is dependent on growth factors (signal 1) and proinflammatory cytokines (signal 2). Since in the context of *T. cruzi* infection, an increase of IFN-γ is absolutely necessary (signal 2), a vaccine should be able to decrease the signal 1 of growth factors to avoid an increase of MDSCs. This hypothesis and the “two-signal” model were supported by the finding that TSf-ISPA-vaccinated group of mice has lower levels of plasma GM-CSF, which correlated with lower levels of MDSCs, allowing an increase in the levels of plasma IFN-γ.

Taken together, the studies performed by depleting MDSCs at day 15 p.i. suggest that those cells are strongly beneficial for parasite persistence, allowing the survival of its host and widely influencing the immune response, even when the mice were previously immunized with a vaccine with a significant protective capacity. The fact that MDSC depletion at day 15 p.i. increased the CD8 effector response but did not cause an improvement in the survival of vaccinated and infected mice, suggests that other strategies would be needed to support the targeting of MDSCs as a valuable tool to enhance the protective capacity of a vaccine candidate. For this purpose, TSf-ISPA vaccinated mice were early treated with 5FU one day before infection and at day 5 p.i. TSf-ISPA-vaccinated mice that were treated with 5FU at the beginning of infection showed higher survival against a high dose of *T. cruzi* than vaccinated mice that did not receive 5FU, supporting that MDSCs could be targeted in a beneficial manner to enhance the protective capacity of a vaccine candidate.

At present, various approaches are under study to delete or inhibit the immunosuppressor arm of the immune system that may act decreasing the efficacy of a vaccine. For instance, molecular inactivators of Tregs or blockers of immunosuppressive cytokines are two strategies under study (Bayry, 2014; Batista-Duharte et al., 2018). In addition, in the field of cancer study, ongoing clinical trials attempt to deplete MDSCs in order to increase vaccine efficacy (Fleming et al., 2018; Liu et al., 2018). However, the strategy to target MDSCs to enhance a vaccine against a pathogen has not been previously addressed. Since we have previously reported that TSf-ISPA immunization caused a slight but significant increase of CD11b+ GR-1+ cells in the spleen (Prochetto et al., 2017), to conclude our study, we sought to evaluate whether 5FU administration at the stage of immunization may enhance our vaccine candidate. TSf-ISPA-immunized mice that were 5FU-treated before each vaccine dose showed a significant increase of survival against a lethal dose of *T. cruzi*, improving the survival of TSf-ISPA-vaccinated mice that were not treated with 5FU. In line with these results, parasitemia levels were also decreased in TSf-ISPA mice that were pretreated with 5FU as compared to immunized mice that did not received 5FU. Although survival is likely the most clear and relevant parameter of protection, parasitemia levels may also have important implications in the context of *T. cruzi* infection. In this sense, a vaccine that decreases parasite load could be useful to avoid the development of chronic Chagas disease and to reduce potential vertical transmission (Bustos et al., 2019).

Several vaccines may cause increases in CD11b+ GR-1+ cells, decreasing the efficacy of the immunization; however, this aspect has received little attention. For instance, it has been reported that the BCG vaccine, the most widely used human vaccine, causes increases in those cells with potential to decrease the vaccine efficacy and even with the capacity to kill DCs (Ribechini et al., 2019). MDSC increases have also been reported in models of *Salmonella* and Simian Immunodeficiency Virus (Cabrera and Marcipar, 2019). Moreover, better protection has also been obtained in those vaccine candidates that decreases MDSCs after immunization or after pathogen challenge, also supporting that MDSCs may be used as a valuable target to be addressed during rational vaccine design (Cabrera and Marcipar, 2019).

Overall, throughout this study we have shown that: a) MDSCs have a wide *in vivo* influence during the acute phase of *T. cruzi* infection, even affecting the immune response of vaccinated mice, influencing DC phenotype as well as cells of both the effector (CD8) and the regulatory arm of the immune system (Tregs); b) although MDSC depletion at day 15 of infection supports that those cells are absolutely necessary for mouse survival, benefiting the parasite, the present results suggest that in the context of vaccination, MDSC could be removed during early infection improving the host survival; c) 5FU treatment at the stage of immunization enhanced the protective capacity of the TSf-ISPA vaccine, supporting the possibility to use this strategy in rational vaccine design not only against *T. cruzi* but also in other pathologies that are characterized by the subversion of the immune system.

## Data Availability Statement

The datasets generated for this study are available on request to the corresponding author.

## Ethics Statement

The animal study was reviewed and approved by Animal Care & Use Committee of the Facultad de Bioquímica y Ciencias Biológicas, UNL.

## Author Contributions

GC and IM designed the experiments. JG and CR performed most of the experiments. EP and IB optimized the protocols and obtained the TSf protein. In addition, they assisted in some experiments. GL produced the ISPA adjuvant and assisted in the experiments. CP and MV designed the experiments and analyzed the data of dendritic cells. AP produced the Tulahuén *T. cruzi* strain used in all the experiments. AP, IM, and GC analyzed the data, designed the figures, and wrote and edited the manuscript. All authors contributed to the article and approved the submitted version.

## Funding

This work was supported by ANPCyT (Argentine National Agency for the Promotion of Science and Technology) (PICT 2018-01164, PICT 2019-01948 and PICT 2015-2544), CONICET (National Scientific and Technical Research Council) and the Universidad Nacional del Litoral, Argentina.

## Conflict of Interest

The authors declare that the research was conducted in the absence of any commercial or financial relationships that could be construed as a potential conflict of interest.

## Abbreviations

*T. cruzi*, *Trypanosoma cruzi*; TS, Trans-sialidase; TSf, Trans-sialidase fragment; Tregs, CD4+ Foxp3+ regulatory T cells; MDSCs, Myeloid-derived suppressor cells.
