# Supplementary material for: Targeting Myeloid-Derived Suppressor Cells to Enhance a Trans-Sialidase-Based Vaccine Against Trypanosoma cruzi
Source: Front Cell Infect Microbiol. 2021 Jul 6;11:671104. doi: 10.3389/fcimb.2021.671104 (PMC8290872; doi:10.3389/fcimb.2021.671104)
Supplement: Supplementary file 1 [file Image_1.pdf]

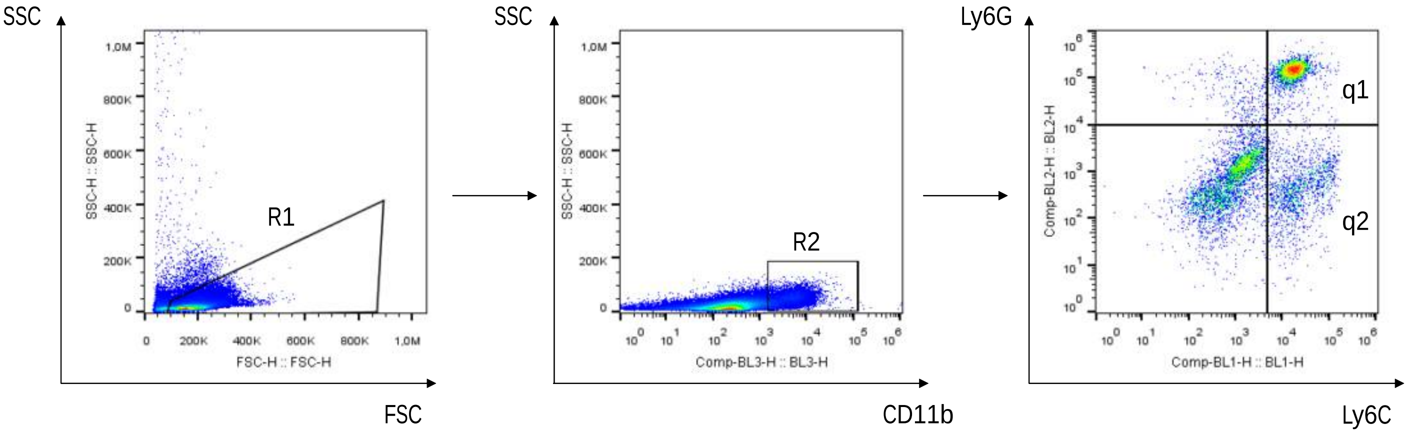

Supplementary Figure 1. Flow cytometry gating strategy for G-MDSCs and M-MDSCs. Spleen cells were stained for Ly6C-FITC, Ly6G-PE and CD11b-PerCPcy5.5.

A wide range of events were selected in the R1 gate by forward scatter (FSC) and side scatter (SSC). CD11b+ cells were then identified within a gate R2 according to CD11b staining and SSC parameter. Finally, Ly6G+ Ly6C+/low (q1) and Ly6G- Ly6C+ (q2) cells were identified within CD11b+ cells (gate R2), using a quadrant strategy.
