# Supplementary material for: Targeting Myeloid-Derived Suppressor Cells to Enhance a Trans-Sialidase-Based Vaccine Against Trypanosoma cruzi
Source: Front Cell Infect Microbiol. 2021 Jul 6;11:671104. doi: 10.3389/fcimb.2021.671104 (PMC8290872; doi:10.3389/fcimb.2021.671104)
Supplement: Supplementary file 2 [file Image_2.pdf]

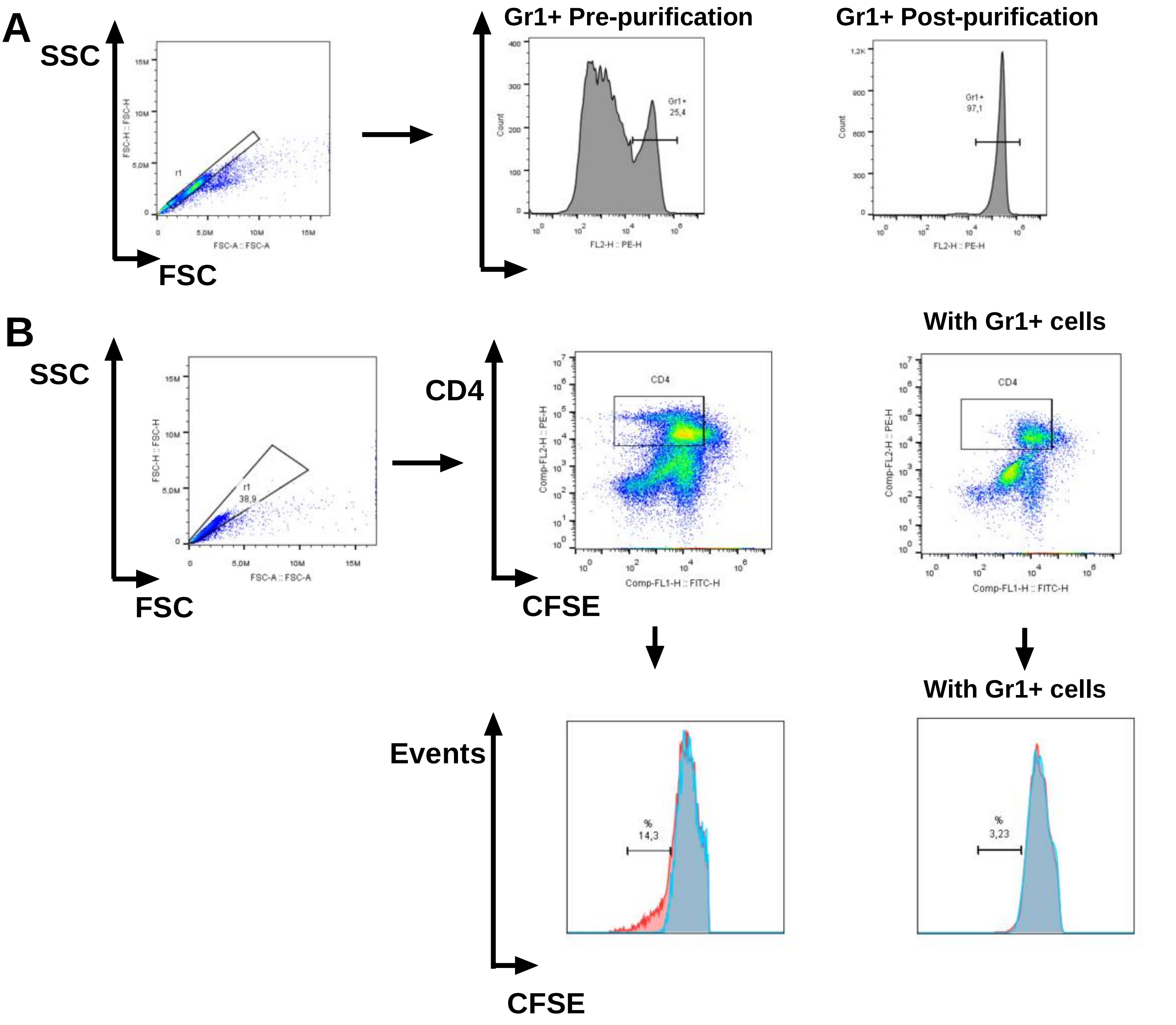

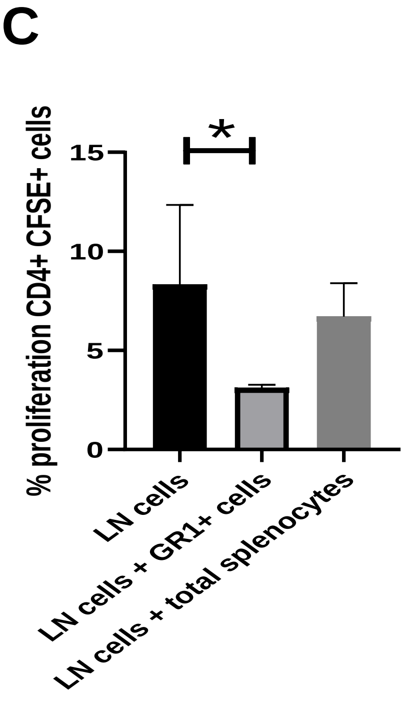

Supplementary Figure 2. MDSC Suppression assay

A) GR-1+ cells were isolated from frozen spleens of PBS-infected mice at day 21 postinfection, using the MDSC purification kit from Miltenyi Biotec and according to manufacturer's instructions. The purity of GR-1+ cells was higher than 96%. B) Total lymph node cells from BALB/c mice were labelled with 10 μM of 5,6-carboxifluorescein diacetate succinimidyl ester (CFSE) (Molecular probes, Eugene, OR) and used as responder cells. Labelled lymph node cells (LN) ($1x10^5$) were cultured in flat-bottom 96-well culture plates (Corning Costar, Cambridge, MA) alone or in a ratio 1:1 with GR-1+ purified cells based on the previous report of Arocena et al. 2014), alternatively, total splenocytes from healthy mice were used instead of GR-1+ cells. Cells were cultured for 72 h at 37 °C and 5% CO_2 in a final volume of 200 uL of RPMI1640 medium (Gibco) supplemented with 10% fetal bovine serum, 2% penicillin (100 μg/mL) and streptomycin (100 U/mL) (Gibco) and 0.4 mM 2-mercaptoethanol. Cell proliferation was induced adding 7,5 ug/ml of Concanavalin A (ConA) and proliferation of CD4+ CFSE+ cells was analyzed by CFSE dilution using overlays with LN cells cultured without conA as reference. C) percentage (%) of CD4+ CFSE+ cell proliferation in: ConA cultured LN cells, conA cultured LN cells in the presence of GR1+ purified cells; and ConA cultured LN cells in the presence of total splenocytes from healthy mice. *p<0,05, Mann-Whitney test, n=4 wells.
