# Supplementary material for: Targeting Myeloid-Derived Suppressor Cells to Enhance a Trans-Sialidase-Based Vaccine Against Trypanosoma cruzi
Source: Front Cell Infect Microbiol. 2021 Jul 6;11:671104. doi: 10.3389/fcimb.2021.671104 (PMC8290872; doi:10.3389/fcimb.2021.671104)
Supplement: Supplementary file 3 [file Image_3.pdf]

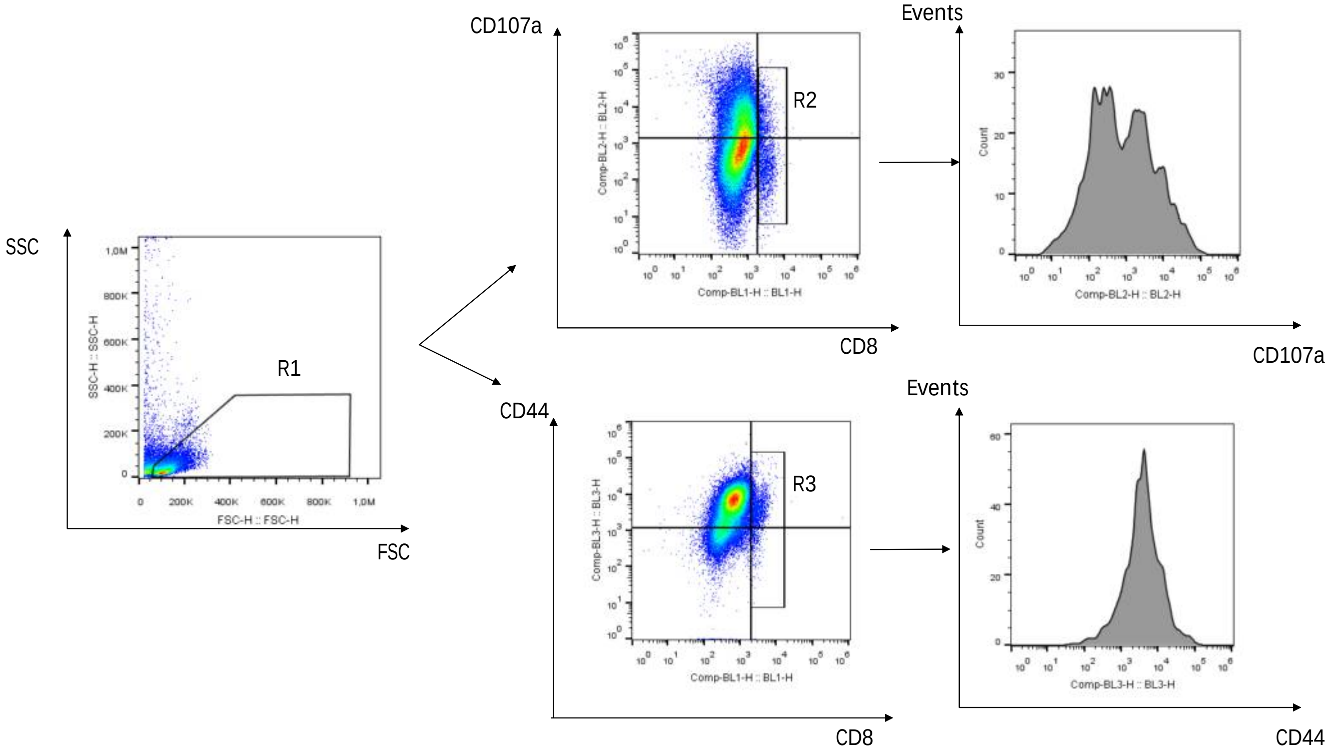

Supplemental Figure 3. Flow cytometry gating strategy for CD8+ CD107a+ and CD8+ CD44+ cells. Spleen cell were stained for CD8-FITC, CD107a-PE and CD44-PerCPcy5.5 A wide range of events were selected in the R1 gate by forward scatter (FSC) and side scatter (SSC). Then, CD8 vs CD107a and CD8 vs CD44 dot plots were created using R1. Quadrant strategy was employed to analyze the percentage of CD107a+ cells in CD8 and CD44+ cells in CD8+ cells. Finally, R2 and R3 gates were used to measure MFI of CD107a+ cells and CD44+ cells in CD8 cells using histograms.
