# Supplementary material for: Targeting Myeloid-Derived Suppressor Cells to Enhance a Trans-Sialidase-Based Vaccine Against Trypanosoma cruzi
Source: Front Cell Infect Microbiol. 2021 Jul 6;11:671104. doi: 10.3389/fcimb.2021.671104 (PMC8290872; doi:10.3389/fcimb.2021.671104)
Supplement: Supplementary file 4 [file Image_4.pdf]

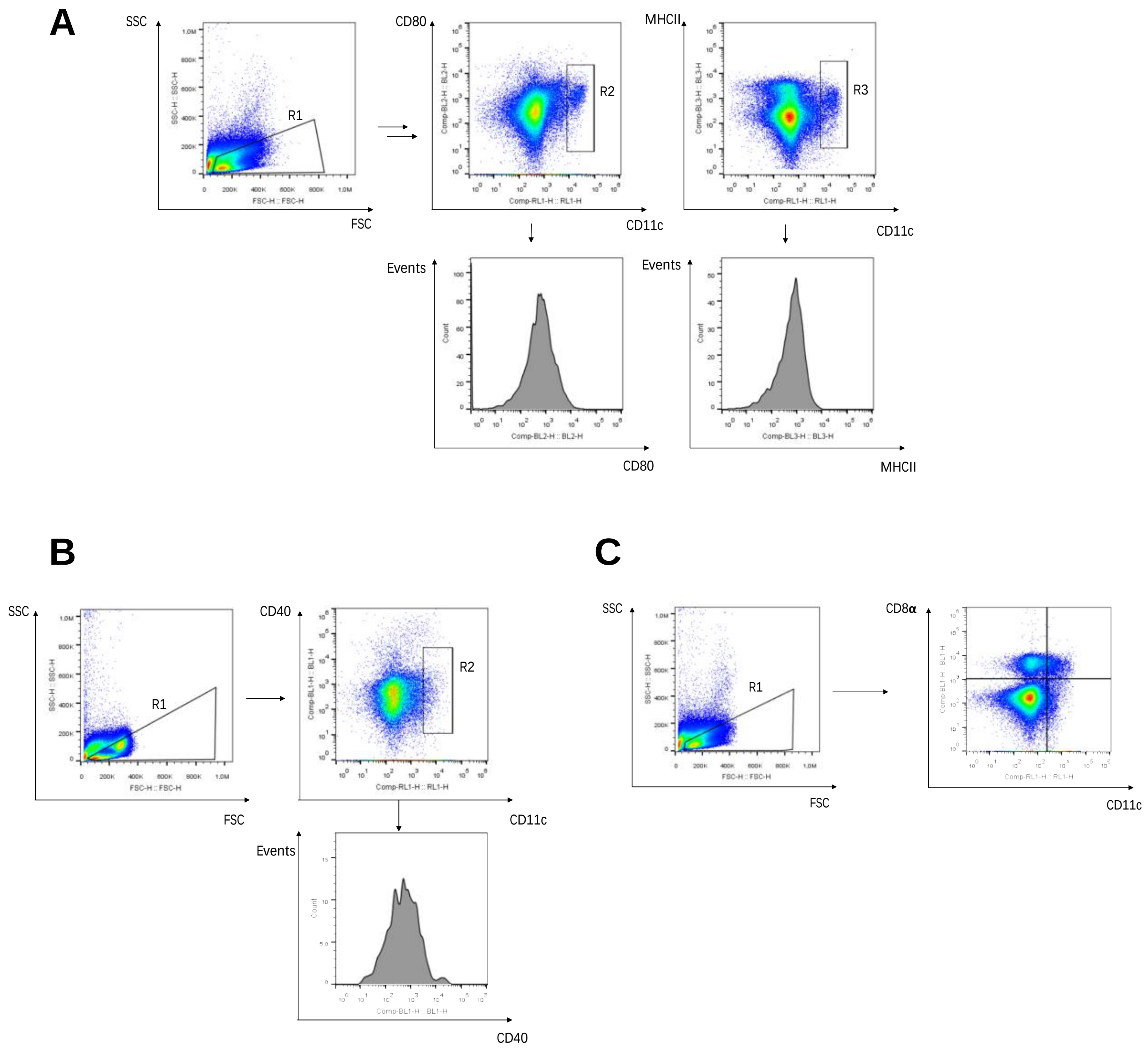

Supplementary Figure 4 Spleen cells were stained for CD11c-APC, CD8-FITC, CD80-PE and MHCII-PerCP-Cy5.5 or CD11c-APC, CD40-FITC and CD80-PE. A) Flow cytometry gating strategy for identifying CD11chigh expressing dendritic cells and CD80 and MHCII expression on those cells. cells. A wide range of events were selected in the R1 gate by forward scatter (FSC) and side scatter (SSC). CD11chigh cells were then identified in a gate R2 or R3 using dot plots based on CD11c vs CD80 or CD11c vs MHCII respectively. Finally, MFI of CD80 or MHCII were analyzed in CD11chigh+ cells using R2 and R3 gates. respectively. (B) A wide range of events were selected in the R1 gate by forward scatter (FSC) and side scatter (SSC). CD11chigh cells were then identified in a gate R2 using dot plots based on CD11c vs CD40. Finally, MFI of CD40 was analyzed in CD11chigh+ cells using the R2 gate. C) A wide range of events were selected in the R1 gate by forward scatter (FSC) and side scatter (SSC). Then, CD11c+ CD8α+ cells were analyzed using the R1 gate and a quadrant strategy.
