# Supplementary material for: Targeting Myeloid-Derived Suppressor Cells to Enhance a Trans-Sialidase-Based Vaccine Against Trypanosoma cruzi
Source: Front Cell Infect Microbiol. 2021 Jul 6;11:671104. doi: 10.3389/fcimb.2021.671104 (PMC8290872; doi:10.3389/fcimb.2021.671104)
Supplement: Supplementary file 5 [file Table_1.docx]

Supplementary Table I.

| FITC | PE | PercP-Cy5.5 | APC |
| --- | --- | --- | --- |
| Figure 1 | | | |
| Ly6C | Ly6G | CD11b |  |
|  | | | |
| Figure 2 | | | |
| iNOS | Ly6G | CD11b |  |
| CD4 |  | Ki-67 |  |
|  | | | |
| Figure 3 | | | |
| CD8 | CD107a | CD44 |  |
|  |  |  |  |
| Figure 4 | | | |
| CD4 | Foxp3 |  |  |
|  | | | |
| Figure 5 | | | |
| CD8 | CD80 | MHCII | CD11c |
| CD40 | CD80 |  | CD11c |

Supplementary Table I. The Figure shows the fluorophores applied and the combinations employed in each Figure of the manuscript.
