# Supplementary material for: Targeting Myeloid-Derived Suppressor Cells to Enhance a Trans-Sialidase-Based Vaccine Against Trypanosoma cruzi
Source: Front Cell Infect Microbiol. 2021 Jul 6;11:671104. doi: 10.3389/fcimb.2021.671104 (PMC8290872; doi:10.3389/fcimb.2021.671104)
Supplement: Supplementary file 6 [file Table_2.docx]

Supplementary Table II

|  | PBS Tc+ | TSf-ISPA Tc+ | PBS 5FU Tc+ | TSf-ISPA 5FU Tc+ |
| --- | --- | --- | --- | --- |
| Percent Survival | 60-100% | 100% | 0% | 60-100% |
| Mean parasitemia | 20-94,5 | 3,2-14,8 | 52-134 | 21-22,8 |

Supplementary table II: Survival and parasitemia of experimental groups of mice. Data correspond to two independent experiments with similar results.

Mice immunized with TSf-ISPA or PBS were treated or not with 5FU and challenged with 900 trypomastigotes of the Tulahuen strain. Percent survival refers to the percent of mice alive at day 21 post infection (p.i.). Mean parasitemia was calculated as the sum of parasites counted in 20 fields in each alive mouse at day 20 p.i./number of alive mouse at day 20 p.i.

(PBS Tc+): PBS-inoculated and infected mice

(TSf-ISPA Tc+): TSf-ISPA-immunized and infected mice.

(PBS Tc+ 5FU): PBS-inoculated, infected and 5FU-treated mice.

(TSf-ISPA Tc+ 5FU): TSf-ISPA-immunized, infected and 5FU-treated mice.
